# The Electrical Conductivity, EMI Absorption Shielding Performance, Curing Process, and Mechanical Properties of Rubber Composites

**DOI:** 10.3390/polym16050566

**Published:** 2024-02-20

**Authors:** Ján Kruželák, Andrea Kvasničáková, Michaela Džuganová, Rastislav Dosoudil, Ivan Hudec, Henrich Krump

**Affiliations:** 1 Department of Plastics, Rubber and Fibres, Faculty of Chemical and Food Technology, Slovak University of Technology in Bratislava, Radlinského 9, 812 37 Bratislava, Slovakia; andrea.kvasnicakova@stuba.sk (A.K.); michaela.dzuganova@stuba.sk (M.D.); ivan.hudec@stuba.sk (I.H.); 2 Department of Electromagnetic Theory, Faculty of Electrical Engineering and Information Technology, Slovak University of Technology in Bratislava, Iľkovičova 3, 812 19 Bratislava, Slovakia; rastislav.dosoudil@stuba.sk; 3 Bizlink Technology, Trenčianska Teplá 1356, 914 01 Trenčianska Teplá, Slovakia

**Keywords:** rubber, manganese–zinc ferrite, nickel–zinc ferrite, carbon black, carbon fibers, electromagnetic absorption shielding

## Abstract

Three types of composites were tested for electromagnetic interference (EMI) absorption shielding effectiveness, the curing process, and their physical–mechanical properties. For the first type of composites, nickel–zinc ferrite, manganese–zinc ferrite, and both fillers in their mutual combinations were incorporated into acrylonitrile–butadiene rubber. The overall content of the filler, or fillers, was kept at 200 phr. Then, carbon black or carbon fibers were incorporated into each rubber formulation at a constant loading of—25 phr, while the content of magnetic fillers was unchanged, at —200 phr. This work focused on the understanding of correlations between the electromagnetic shielding parameters and electrical conductivity of composites in relation to their EMI absorption shielding effectiveness. The absorption shielding abilities of materials were evaluated within a frequency bandwidth from 1 MHz to 6 GHz. This study revealed good correlation among permittivity, conductivity, and EMI absorption effectiveness. Although the absorption shielding efficiency of composites filled only with ferrites seems to be the highest, the absorption maxima of those composites reached over 6 GHz. The application of carbon-based fillers resulted in the higher electrical conductivity and higher permittivity of composites, which was reflected in their lower absorption shielding performance. However, the composites filled with ferrites and carbon-based fillers absorbed electromagnetic radiation within the desired frequency range. The presence of carbon-based fillers caused improvement in the tensile behavior of composites. This study also demonstrated that the higher the ratio of nickel–zinc ferrite in combined magnetic fillers, the better the absorption shielding efficiency.

## 1. Introduction

Rapid development in radio and telecommunication devices has created a new kind of environmental pollution, which is associated with the accumulation of electromagnetic radiation in the environment. This radiation is called electromagnetic smog, or electromagnetic interference (EMI). The accumulation of EMI in the environment has raised serious concern, as electromagnetic radiation waves can not only disturb the normal operation of electronic equipment or cause it to malfunction but also pose a serious threat to human physiological function [1,2,3,4]. Thus, the development of materials with EMI shielding performance has become more and more desirable.

Rubber matrices are electrical insulators, and, thus, they do not provide shielding effects. However, the incorporation of suitable fillers (in the form of particles, fibers, sheets, tubes, platelets, etc.) imparts EMI shielding performance to them. Different types of fillers based on carbon (carbon black, carbon fibers, carbon nanotubes, graphite, or graphene), as well as inorganic fillers (metals, metal oxides, MXenes, ferrites), have been tested for this purpose [5,6,7,8,9,10,11,12]. Although the rubber matrix is the carrier of the main characteristics, the type and the content of the filler can have significant influences on process-ability, physical–mechanical or dynamic properties, and EMI shielding performance. Carbon-based fillers reinforce the rubber matrix and provide good physical–mechanical properties to rubber composites. They also enhance thermal flow and electrical conductivity through composite materials. The disadvantage of conductive materials used for EMI shielding is that they have a different impedance from that of the ambient environment in which electromagnetic waves proliferate. This impedance mismatch at the interfacial area between the ambient environment and the shield results in a high proportion of radiation being reflected from the shielding material [13,14,15]. Reflection of EMI is unwanted, as the reflected radiation can interfere with other radiation sources, thus causing secondary EMI effects. 

On the other hand, materials having magnetic dipoles, such as ferrites, reduce the difference between the impedance of the shield and the ambient environment, which results in the attenuation of EMI by absorption [16,17,18]. The radiation absorbed by the shield is usually transferred into heat by the Joule effect. The absorption of the radiation by the shield is the most efficient way to reduce EMI. However, the incorporation of solid ferrite powdery fillers into rubber matrices usually leads to the deterioration of physical–mechanical and utilitarian properties. 

Thus, the combination of carbon-based fillers and materials having magnetic dipoles seems to be a good way to fabricate rubber materials with good shielding efficiency and utilitarian properties [19,20,21,22]. Rubber composites exhibit good elasticity, flexibility, dimensional stability, and corrosion resistance; they are also lightweight and low cost. 

Generally used electronic appliances emit electromagnetic radiation at frequencies below 4–5 GHz. Our previous experimental works have revealed that, for low-frequency absorption shielding, magnetic soft ferrites are good candidates for the manufacturing of rubber composites. Manganese–zinc ferrite and nickel–zinc ferrite belong to the well-established ferrite family. Both ferrites have been incorporated into rubber matrices in concentration scales from 100 phr to 500 phr. It has been shown that the best absorption shielding performance was demonstrated by composite materials containing 200 phr of ferrites. Thus, in this work, nickel–zinc ferrite, manganese–zinc ferrite, and mutual combinations of both ferrites were kept at a constant loading of—200 phr. In the following two series of rubber compounds, carbon-based fillers—carbon black or carbon fibers—were additionally added into each composite at a constant amount of—25 phr. Acrylonitrile–butadiene rubber (NBR) was used as a rubber matrix. NBR is one of the most widely used specialty types of rubber, with good correlation between cost and properties. Due to its polar character, it has good resistance to oils and non-polar solvents. This work focused on the investigation of ferrites and combinations of ferrites with carbon-based fillers as well as their influences on the absorption shielding efficiency of composites. The curing process and physical–mechanical properties were also examined. 

## 2. Experimentation

### 2.1. Materials

In this work, nickel–zinc ferrite, NiZn, and manganese–zinc ferrite, MnZn, were used as magnetic soft ferrites. Both ferrites represent commercially available powdery fillers and were provided by the Epcos Company, Šumperk, Czech Republic. They exhibit a spinel-type structure, and the particle size distributions of the ferrites were very similar. They ranged from 0.2 to 70 μm for NiZn ferrite and from 0.7 to 50 μm for MnZn ferrite. The particle size distributions of both fillers, with parameters D10 and D50, are summarized in Table 1. D10 and D50 demonstrate ratios of particle size lower than the respective given values. D50 is the median, showing that 50% of the particles were smaller than around 21 μm for NiZn ferrite and smaller than roughly 16 μm for MnZn filler. 

As carbon-based fillers, carbon fibers, CF, and carbon black, CB, were used. Specially conductive carbon black, under the trade name VULCAN^®^ XC 72, was supplied by the Cabot Corporation, Boston, MA, USA. Carbon fibers with the commercial trademark Carbiso^TM^G were provided by ELG Carbon Fibre Ltd., Bilston, UK. The lengths of fibers ranged between 80 and 100 μm, with a fiber diameter of 7.5 μm. Acrylonitrile–butadiene rubber, NBR (SKN 3345, content of acrylonitrile = 31–35%), provided by Sibur International, Moscow, Russia, served as a rubber matrix. Sulfur, in combination with activators (stearic acid and zinc oxide) and an accelerator (N-cyclohexyl-2-benzothiazole sulfenamide CBS), was used for the cross-linking of rubber compounds. The chemicals for the sulfur vulcanization process were provided by Vegum a.s., Dolné Vestenice, Slovak Republic. 

### 2.2. Methods

#### 2.2.1. Fabrication and Curing of Rubber Compounds

Three types of composite materials were fabricated and examined for their properties. In the first type of composites, MnZn ferrite, NiZn ferrite, and mutual combinations of both ferrites were applied into an NBR-based rubber matrix. The total content of magnetic fillers was kept at a constant level—200 phr; only the mutual ratio of both fillers was uniformly changed. The composition of materials and their designation are mentioned in Table 2. In the second and third types of composites, carbon black or carbon fibers, respectively, were added at a constant loading—25 phr, while the content of magnetic fillers was also kept at a constant level. The composition of hybrid composites is summarized in Table 3. In graphical illustrations, the composites filled only with ferrites are designated as NBR; hybrid composites based on CB or CF and ferrites are marked as NBR/CB or NBR/CF, respectively. 

The composites were fabricated in a Bradender laboratory kneader (Brabender GmbH & Co. KG, Duisburg, Germany) in a two-step mixing process. The temperature of mixing was set to 90 °C with a rotor revolution frequency of 55 rpm. First, the rubber was put into the chamber and masticated for 2.5 min. Then, stearic acid and zinc oxide were added and after 2 min, ferrite or ferrites in combination were applied. The mixing process continued for 4.5 min and after that, the compounds were cooled down and sheeted in a two-roll mill. The sulfur and accelerator were added in the second step and the mixing process proceeded for 4 min at 90 °C and 55 rpm. Finally, the compounds were additionally homogenized and sheeted in a two-roll mill. To fabricate hybrid composites, that is, the composites filled with carbon-based fillers and ferrites, rubber batches based on carbon black or carbon fibers were first pre-compounded in a Buzuluk large-volume kneader (Buzuluk Inc., Komárov, Czech Republic). Then, the compounding procedure with magnetic fillers and curing additives proceeded following the same procedure as mentioned above. 

The rubber compounds were subsequently cured at 160 °C and a pressure of 15 MPa into thin sheets of 15 × 15 cm and 2 mm thickness. A Fontijne low platen hydraulic press (Fontijne Presses, Turbineweg, The Netherlands) was used for the vulcanization process and the time of heating corresponded to the optimum cure time of each rubber formulation. 

#### 2.2.2. Determination of Curing Characteristics

Curing characteristics were evaluated from corresponding curing isotherms of rubber compounds, which were experimentally measured in an MDR 2000 oscillatory rheometer (Alpha Technologies, Akron, OH, USA).

The investigated curing parameters were:

*M_L_* (dN·m)—minimum torque;

*M_H_* (dN·m)—maximum torque;

*t_c_*_90_ (min)—optimum cure time;

*t_s_*_1_ (min)—scorch time;

*R_v_* (min^−1^)—cure rate index, defined as:

(1) $$R_{v}=\frac{100}{t_{c90}-t_{s1}}$$

#### 2.2.3. Investigation of Mechanical Characteristics

A Zwick Roell/Z 2.5 instrument (Zwick GmbH & Co. KG, Ulm, Germany) was used to determine the tensile properties of composites. The cross-head speed of the measuring device was set to 500 mm.min^−1^ and the tests were performed in accordance with the technical standards. Dumbbell-shaped test samples (width = 6.4 mm, length = 80 mm, thickness = 2 mm) were used for measurements. The hardness was measured by using a durometer and was defined in Shore A.

#### 2.2.4. Investigation of Shielding Characteristics

The frequency dependencies of complex (relative) permeability µ *=* µ′ − jµ″ for toroidal samples were measured using a combined impedance/network analysis method by means of a vector analyzer (Agilent E5071C, Agilent Technologies, Santa Clara, CA, USA) in the frequency range of 1 MHz–6 GHz. During measurements, a toroidal sample was inserted into a magnetic holder (Agilent 16454A) and the complex permeability was evaluated from measured impedances (1):

μ = μ′ − jμ″ = 1 + (Z − Z_air_)/(jhμ_0_ f ln(b/c)) (2)

where Z and Z_air_ are the input complex impedances of the 16454A holder with and without a toroidal sample, respectively, h is the height of the sample, μ_0_ = 4π · 10^−7^ H/m is the permeability of free space, f is the frequency, and b and c are the outer and inner diameters of the sample.

The frequency dependencies of complex (relative) permittivity ε *=* ε′ − jε″ for disc samples were measured using a combined impedance/network analysis method by means of a vector analyzer (Agilent E5071C) in the frequency range of 1 MHz–6 GHz. During measurements, a disc sample was inserted into a dielectric holder (Agilent 16453A) and the complex permittivity was computed from measured admittance (2):

ε = ε′ − jε″ = (Y · h)/(jωε_o_S) (3)

where *Y* is the input complex admittance of the 16453A holder with a disc sample, h is the height of the sample, ε_0_ = 8.854 · 10^−12^ F/m is the permittivity of free space, and S is the area of the lower electrode. In case of electrically conductive material with *dc* electrical conductivity *σ_dc_*, the imaginary part of ε should be replaced by ε″ − *σ_dc_*/2πε_o_. The electrical *dc* conductivity of composite materials was evaluated using a standard two-probe method.

High-frequency single-layer electromagnetic wave absorption properties (return loss RL, matching thickness *d_m_*, matching frequency f*_m_*, bandwidth Δf for RL at −10 dB and RL at −20 dB, and the minimum of return loss RL*_min_*) of composite materials were obtained by calculations of return loss (3):

RL = 20 log |(Z_in_ − 1)/(Z_in_ + 1)| (4)

where Z_in_ = (μ/ε)^1/2^tanh[(jω·*d*/*c*)(μ·ε)] is the normalized value of the input complex impedance of the absorber, *d* is the thickness of the single-layer absorber (backed by a metal sheet), and c is the velocity of light in vacuum. The composite absorbs maximum electromagnetic plane-wave energy when the normalized value of impedance Z_in_ ≈ 1. The maximum absorption is then reached at a matching frequency f = f*_m_*, matching thickness *d* = *d_m_*, and minimum return loss RL*_min_*. The thickness of the samples for determination of electromagnetic parameters was 2 mm. 

## 3. Results and Discussion

### 3.1. Curing Process

**Figure 1:**
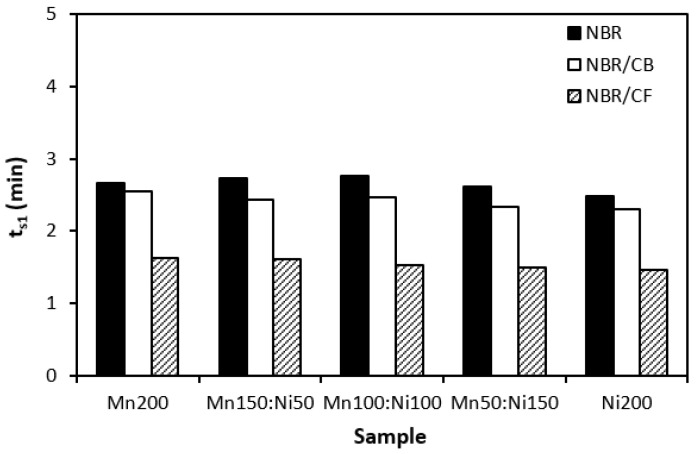
Influence of fillers on scorch time *t_s_*_1_ of rubber compounds.

**Figure 2:**
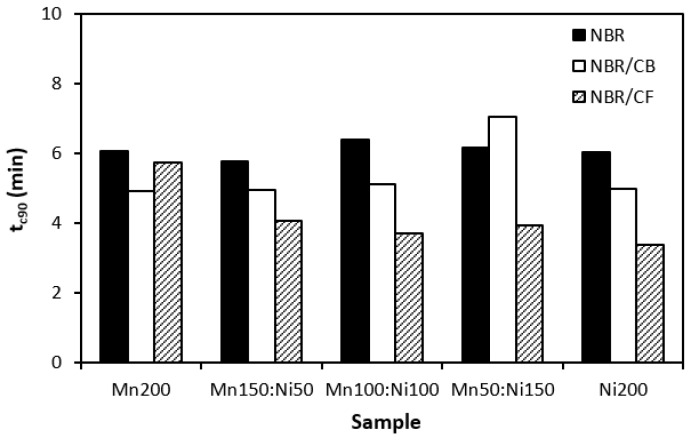
Influence of fillers on optimum cure time *t_c_*_90_ of rubber compounds.

**Figure 3:**
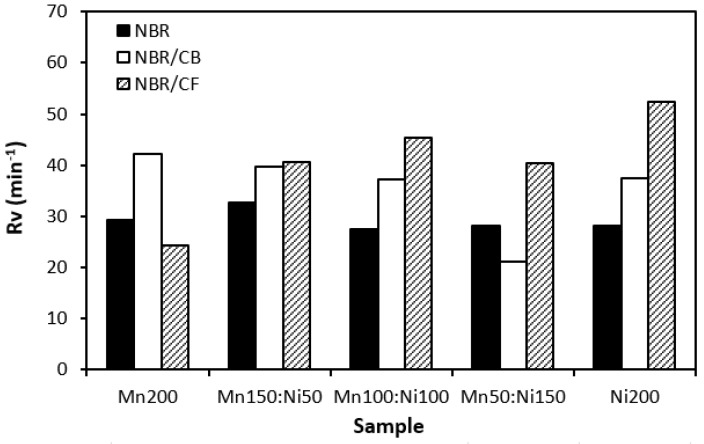
Influence of fillers on curing rate index *Rv* of rubber compounds.

**Figure 4:**
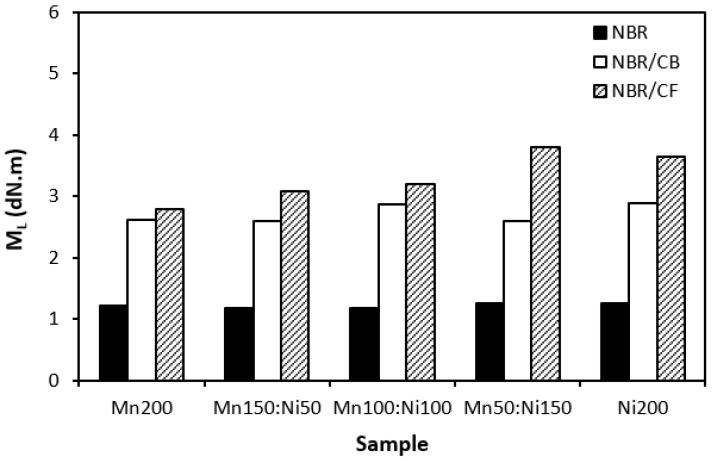
Influence of fillers on minimum torque *M_L_* of rubber compounds.

**Figure 5:**
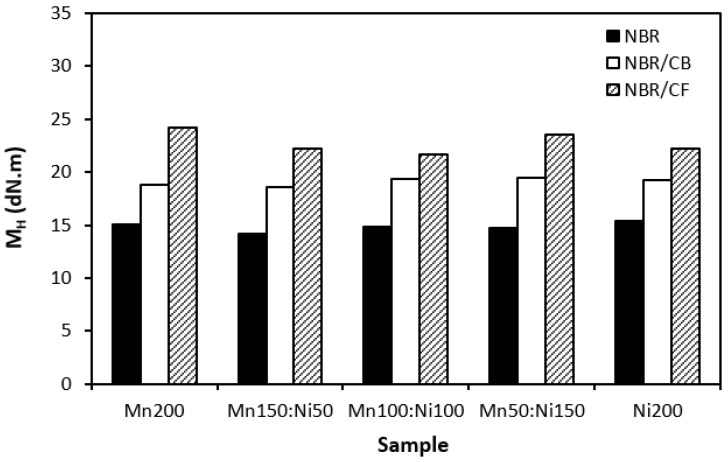
Influence of fillers on maximum torque *M_H_* of rubber compounds.

The influence of the fillers on the vulcanization process of rubber formulations was evaluated by investigation of curing characteristics, scorch time *t_s_*_1_, optimum cure time *t_c_*_90_, cure rate index *R_v_*, minimum torque *M_L_*, and maximum torque *M_H_*. As shown in Figure 1, the longest scorch time exhibited rubber compounds filled with ferrites. The application of carbon-based fillers resulted in a decrease in scorch time. The lowest scorch time exhibited rubber compounds filled with ferrites and carbon fibers. As seen, there was almost no change in scorch time recorded dependent on the type of ferrite or ferrite combination. A similar statement can be applied to the optimum cure time (Figure 2). The longest optimum cure time demonstrated rubber compounds filled with ferrites, while the shortest time needed for optimum cross-linking required those based on ferrites and carbon fibers. Although, the *t_c_*_90_ of the formulations filled with ferrites and CF seems to have a decreasing trend with an increasing proportion of NiZn filler; overall, it can be said that no significant influence of magnetic fillers on optimum cure time was observed. The shortest scorch time as well as optimum cure time of the materials based on ferrites and CF were reflected in their highest cure rate index (Figure 3). That means the curing process of the materials with incorporated carbon fibers proceeded the fastest. On the other hand, the lowest curing kinetics were found for the compounds filled only with magnetic fillers. Based on the obtained results, it becomes apparent that the presence of carbon-based fillers resulted in the acceleration of the curing process. Carbon-based fillers are characterized by unique electrical as well as thermal conductivities. Their incorporation into the rubber matrix enhanced thermal conductivity and thermal flow through the materials, which subsequently promoted faster vulcanization. From Figure 4 and Figure 5, it is shown that the lowest minimum as well as maximum torque were found for the rubber compounds filled only with ferrites. The application of carbon-based fillers resulted in increases in both minimum and maximum torques. The highest values of both parameters were found for the compounds filled with ferrites and CF. Again, we recorded no significant influence of the type of ferrite or ferrites in their mutual combinations on M_L_ and M_H_. The minimum torque corresponds to the viscosity of rubber compounds before the curing process starts, which clearly points out to the increase in the viscosity by incorporation of carbon-based fillers. This can be attributed to the higher viscosity of the fillers than that of the rubber matrix. Also, small nano-sized filler particles (like carbon black or carbon fibers) contribute to an increase in viscosity much higher than that of micro-sized magnetic fillers. The maximum torque relates to the viscosity of cured rubber compounds, which is then closely connected with the cross-link density. Carbon-based fillers have much higher compatibility with the rubber matrix, and very good adhesion between the matrix and the carbon fillers on the interface is formed. The thin rubber layer on the rubber recorded filler interface is so strongly bonded to fillers by physical adsorption or chemisorption that it behaves as a polymer in a glassy state and markedly contributes to the increases in viscosity and cross-link density.

### 3.2. Electromagnetic Absorption Parameters and Electrical Conductivity

Generally used electronic equipment like TV sets, laptops, mobile phones, etc., emit electromagnetic radiation within low-frequency ranges, usually between 1–4 GHz. Thus, the shielding of EMI within this frequency bandwidth is of high importance. As already mentioned, shielding by the reflection mechanism is often ineffective, as electromagnetic plane waves reflect from the surface of the shield but still propagates through the ambient environment. By contrast, absorption of electromagnetic radiation is the most promising way to protect the utility of electronic equipment and human health. Therefore, the current work deals with the investigation of the absorption shielding efficiency of composites, which means the ability of materials to absorb electromagnetic radiation. The absorption shielding performance of composites was evaluated within the frequency bandwidth from 1 MHz to 6 GHz, which covers the operation frequency of commonly used electronic and electromagnetic instrumentation. The electromagnetic parameters complex permittivity and complex permeability were first determined. The complex permeability consists of two parts, real µ′ and imaginary µ″ permeability. The real part corresponds to magnetic storage capacity, while imaginary permeability represents magnetic dissipation or losses. 

**Figure 6:**
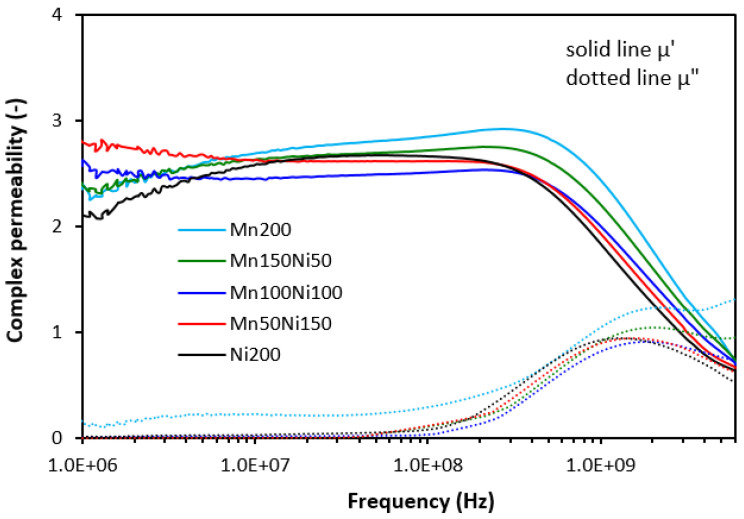
Frequency dependences of complex permeability for composites filled with ferrites.

**Figure 7:**
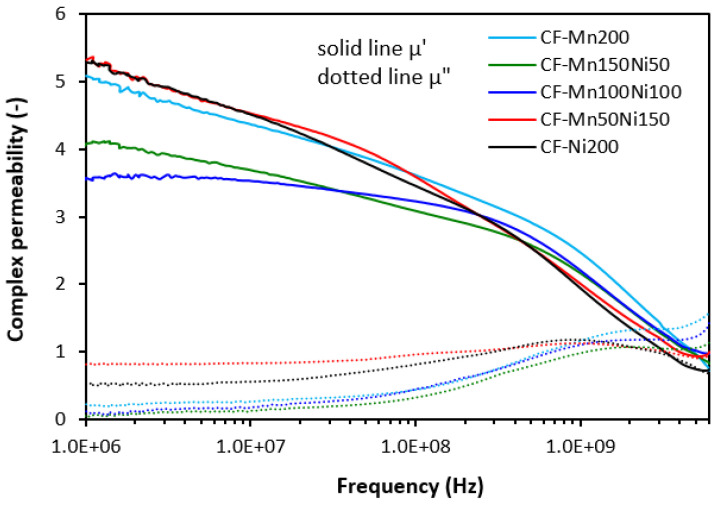
Frequency dependences of complex permeability for composites filled with ferrites and carbon fibers.

**Figure 8:**
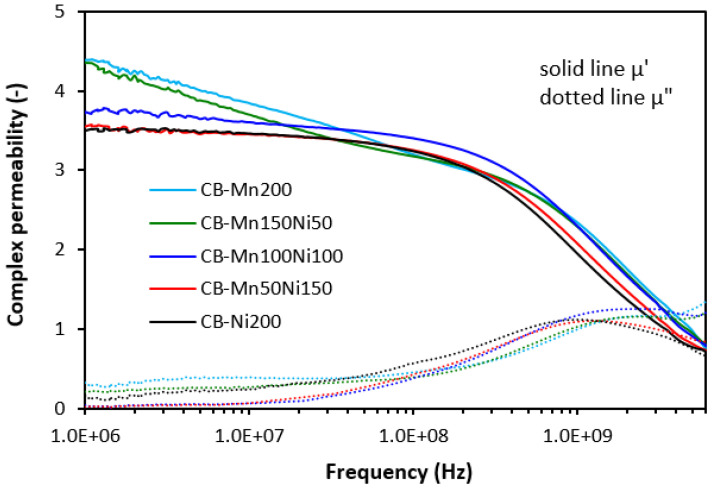
Frequency dependences of complex permeability for composites filled with ferrites and carbon black.

The frequency dependences of complex permeability for tested composites are graphically illustrated in Figure 6, Figure 7 and Figure 8. In Figure 6, it is shown that the real permeability of composites filled only with ferrites does not change with frequency up to roughly 500 MHz, then it significantly drops down (under 1). The highest µ′ seems to be associated with the composite filled with 200 phr of MnZn ferrite, although it can be stated that no significant changes in real permeability were recorded in dependence on the type of ferrite or ferrites in their mutual combinations. Very low influence of fillers’ composition was also recorded for imaginary permeability µ″, which was frequency-independent up to 100 MHz. Then, it reached the maximum (between 1–2 GHz), which corresponds to maximum magnetic dissipation (resonance frequency), and dropped down. 

The frequency dependences of complex permeability for composites filled with ferrites and carbon fibers (Figure 7) or ferrites and carbon black (Figure 8) were very similar. The real part showed a slight decreasing trend with an increase in frequency to 300–500 MHz. Then, a sharp decrease occurred up to a maximum frequency. The imaginary permeability did not change very much with frequency to roughly 100–200 MHz. It reached the maximum dissipation at a resonance frequency (1–2 GHz). The real and imaginary parts of hybrid composites seemed to be slightly higher when compared to equivalent composites filled with ferrites, mainly at very low frequencies. With frequency increase, differences between permeabilities became negligible and no meaningful influence of the tested fillers on complex permeability was observed. 

**Figure 9:**
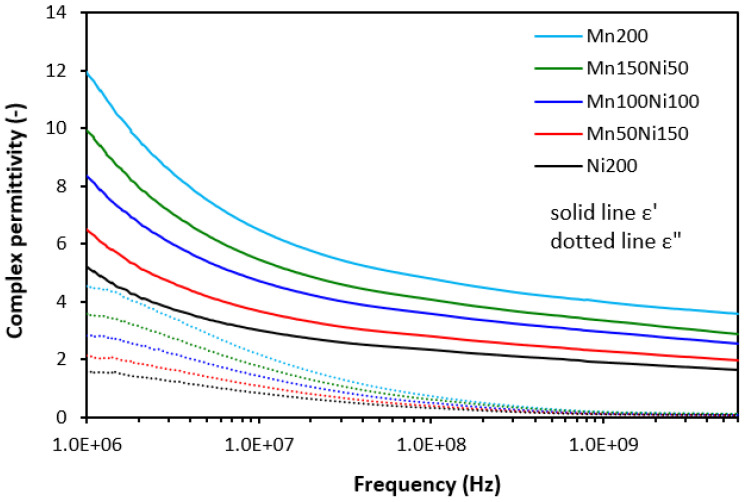
Frequency dependences of complex permittivity for composites filled with ferrites.

On the other side, as shown in Figure 9, we recorded a clear influence of materials’ complex permittivity on the type of ferrite or ferrites in their mutual combinations. The highest value of the real part was manifested in the composite filled with manganese–zinc ferrite (Mn200), while the lowest one was found for the composite filled with nickel–zinc ferrite (Ni200). It can be stated that the higher the amount of nickel–zinc ferrite in magnetic filler combinations, the lower the ε′. The highest differences between the real permittivity were observed at the initial frequency (ε′ = 12 for the composite Mn200, ε′ = 5.2 for the composite Ni200 at 1 MHz). With an increase in frequency, the differences in the real part became smaller. From Figure 9, it is also shown that upon the first strong decline of ε′ at low frequencies, the continual decreasing trend was recorded with the next frequency increase. The recorded values were ε′ = 3.6 and ε′ = 1.6 at 6 GHz for the composites Mn200 and Ni200, respectively. The imaginary permittivity was lower than the real part and was also found to be dependent on the type of ferrite or magnetic fillers in their combinations, mainly at low frequencies. With an increase in frequency, the ε″ declined to a very low value with almost no influence on the frequency or magnetic fillers. 

**Figure 10:**
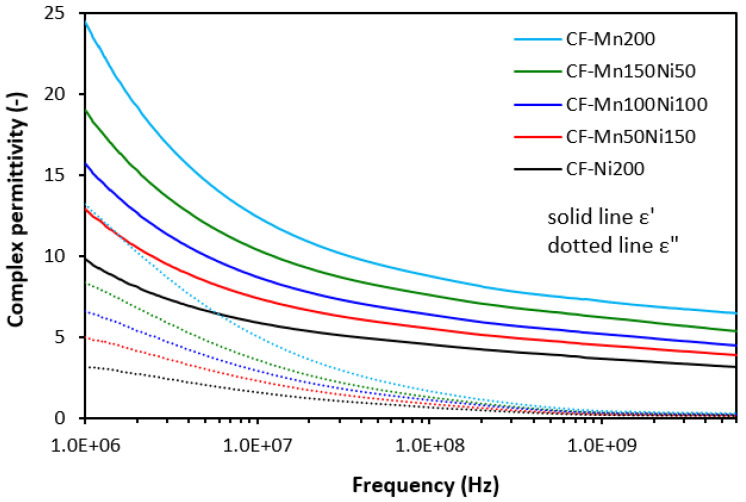
Frequency dependences of complex permittivity for composites filled with ferrites and carbon fibers.

**Figure 11:**
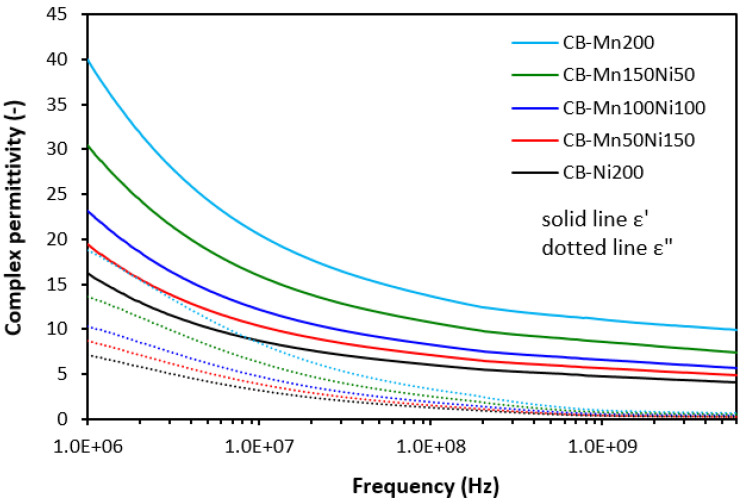
Frequency dependences of complex permittivity for composites filled with ferrites and carbon black.

The complex permittivity of hybrid composites was also strongly dependent on ferrite or ferrites in their combinations, meaning that the higher the amount of nickel–zinc ferrite, the lower the real and imaginary permittivity values (Figure 10 and Figure 11). The higher the frequency, the lower both ε′ and ε″ are. When comparing the complex permittivity of composites, it becomes apparent that the application of carbon-based fillers resulted in the enhancement of both the real and imaginary parts. The calculated value of ε′ was 24.5 for the composite with designation CF-Mn200 at 1 MHz (Figure 10). Upon an increase in frequency up to the maximum, the real part declined to 6.5. The real part of the composite CF-Ni200 decreased from 9.8 at 1 MHz to 3.2 at 6 GHz. The highest complex permittivity was found for composites filled with a combination of ferrites and carbon black (Figure 11). The real part of the composite CB-Mn200 reached almost 40 at 1 MHz, followed by a decrease to 10 at 6 GHz. The imaginary permittivity decreased from 18.7 down to 0.6 when the frequency increased from 1 MHz up to its maximum value. The composite CB-Ni200 exhibited real and imaginary parts of 16.2 and 7.2 at 1 MHz, respectively, which decreased down to ε′ = 4.1 and ε″ = 0.2 at a maximum frequency. 

The absorption shielding efficiency of composites was investigated based on previously calculated parameters. Absorption shielding efficiency of composites was characterized through determination of return loss *RL*. Return loss provides information about the amount of EMI, which is absorbed by the composite shield. Materials exhibiting return loss at −10 dB can absorb about 90–95% of incident radiation plane waves. Lower return loss relates to higher EMI absorption. Material shields exhibiting return loss at −20 dB have been reported to absorb almost 99% of harmful EMI and, thus, they are excellent radiation absorbers [23,24,25]. The efficiency of absorption shielding depends on frequency bandwidths. This means the broader the frequency bandwidths for absorption shielding, the higher the absorption shielding ability of the materials. 

**Figure 12:**
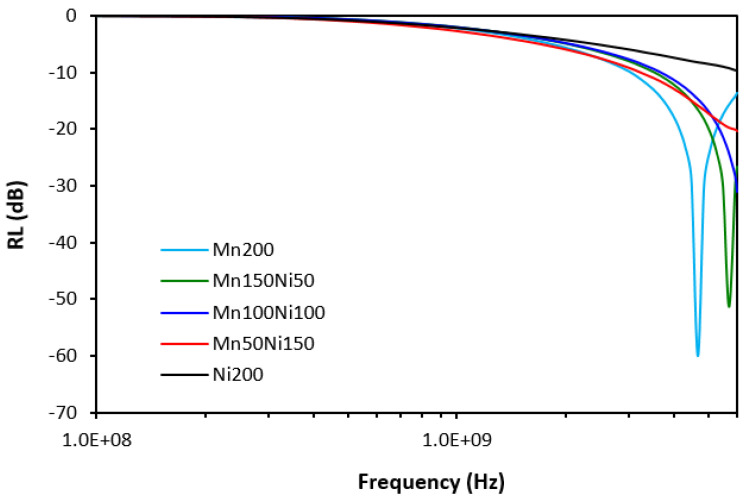
Frequency dependences of return loss for composites filled with ferrites.

The frequency dependences of return loss *RL* for composites filled with ferrites or their mutual combinations are graphically illustrated in Figure 12. The calculated values of their electromagnetic absorption characteristics are summarized in Table 4. RL*_min_* represents the minimum value of return loss at a matching frequency or maximum absorption shielding efficiency, f*_m_* is the matching frequency, and Δf at −10 dB and −20 dB summarizes the effective frequency absorption bandwidth of composites at the given return loss *RL*. It must be noted that composites with an equivalent ratio of both ferrites (Mn100Ni100) and composites with designations Mn50Ni150 and Ni200 reached absorption maxima over the tested frequency of 6 GHz. The electromagnetic absorption parameters summarized in Table 4 represent only the results calculated to the maximum frequency—6 GHz. The composite filled only with nickel–zinc ferrite did not reach return loss even at −10 dB within the tested frequency range and, thus, there are no data of electromagnetic absorption parameters available for this composite. It can be inferred that the higher the ratio of NiZn ferrite in magnetic fillers combinations, the higher the frequency at which the composites provided absorption shielding efficiency. Within the tested frequency range, the evident absorption maxima were recorded only for the composite filled with 200 phr of manganese–zinc ferrite (Mn200) and for the composite Mn150Ni50. The absorption maximum of the sample Mn200 was −60 dB at a matching frequency 4670 MHz, while the composite Mn150Ni50 reached an absorption maximum of −51.4 dB at a matching frequency of 5700 MHz. The effective absorption frequency bandwidth of the composite filled with manganese–zinc ferrite ranged from 3050 MHz to 6 GHz at RL = −10 dB and from 4150 MHz to 5250 MHz at RL = −20 dB. The effective frequency bandwidth for the composite Mn150Ni50 moved from 3550 MHz at −10 dB and from 5020 MHz at −20 dB to 6 GHz. 

**Figure 13:**
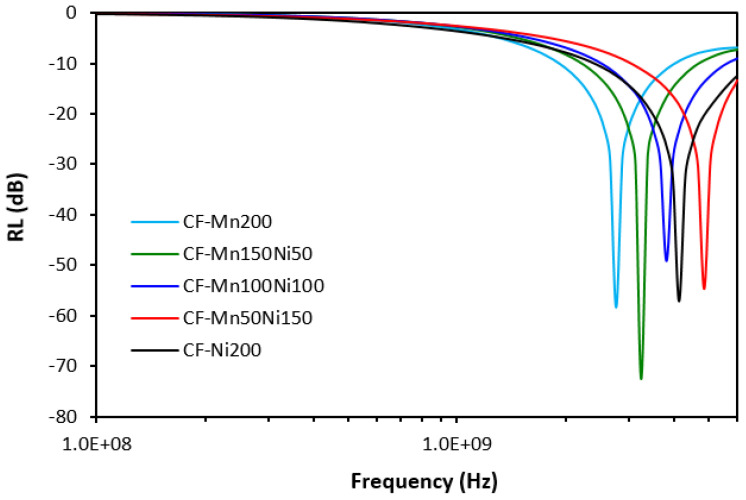
Frequency dependences of return loss for composites filled with ferrites and carbon fibers.

From Figure 13, it becomes apparent that the combination of magnetic fillers with carbon fibers resulted in the shifting of EMI absorption shielding efficiency to lower frequencies and all composites exhibited clear absorption peaks within the tested frequency range. As also shown in Figure 13 and Table 5, the absorption shielding performance was clearly dependent on the type of ferrite or ferrites in their combinations. The composite filled with carbon fibers and manganese–zinc ferrite (CF-Mn200) showed absorption shielding performance at the lowest frequency (matching frequency was 2769 MHz with minimum value of return loss −58.3 dB). This composite exhibited the narrowest effective frequency bandwidth at RL = −10 and −20 dB (from 1920 MHz to 4050 MHz at −10 dB and from 2500 MHz to 3100 MHz at −20 dB). Thus, it can be stated that this composite is the worst absorber of electromagnetic radiation. With increasing amounts of NiZn ferrite, the absorption shielding performance shifted to higher frequencies. The composite filled with CF and 200 phr nickel–zinc ferrite (CF-Ni200) absorbed electromagnetic radiation at the highest frequency (f*_m_* = 4140 MHz, RL*_min_* = −57 dB). The effective frequency bandwidth ranged from 2.4 GHz to 6 GHz at −10 dB (Δf = 3600 MHz) and from 3500 MHz to 4880 MHz at −20 dB (Δf = 1380 MHz). The broadest absorption bandwidths suggest that this composite is the most effective absorber of electromagnetic radiation. When looking at Figure 13 and Table 5, one can see that effective frequency bandwidths of composites became broader with increasing proportions of NiZn ferrite. Thus, it can be concluded that nickel–zinc ferrite exhibits better absorption shielding performance. 

**Figure 14:**
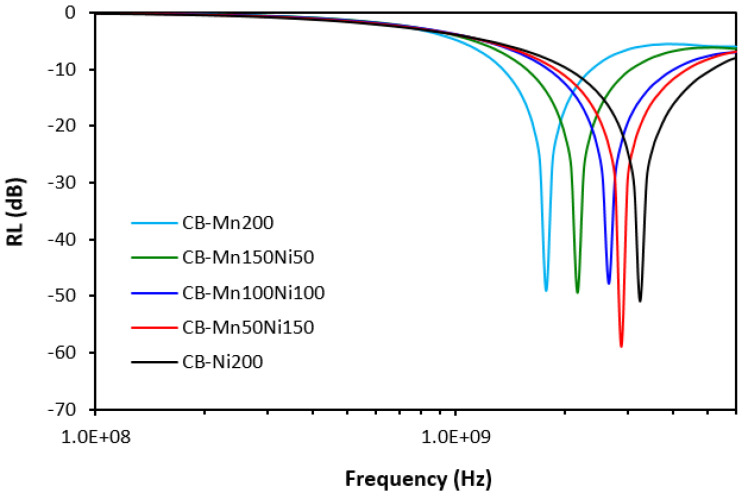
Frequency dependences of return loss for composites filled with ferrites and carbon black.

In Figure 14 and Table 6, it is shown that the frequency dependences of composites filled with ferrites and carbon black are also strongly influenced by the type of ferrite or ferrites in their combinations. Again, with an increasing content of NiZn ferrite, the absorption maxima shifted to higher frequencies and the effective frequency bandwidth at −10 and −20 dB became broader. The absorption maximum for the composite filled with CB and 200 phr of manganese–zinc ferrite (CB-Mn200) was −49 dB at 1780 MHz, while the composite with designation CB-Ni200 demonstrated an absorption maximum of −51 dB at 3250 MHz. The composite CB-Mn200 exhibited the narrowest absorption peak (RL ranged between 1340 MHz–2400 MHz at −10 dB and from 1630 MHz to 1930 MHz at −20 dB). On the other hand, the widest effective frequency bandwidths of the composite CB-Ni200 was between 2020 MHz–5120 MHz (Δf = 3100 MHz) and between 2840 MHz–3720 MHz (Δf = 880 MHz) at −10 and −20 dB, respectively, rank this composite as the best absorber of EMI. 

When comparing electromagnetic absorption parameters of composites filled only with ferrites (considering only the composites with designations Mn200 and Mn150Ni50, which demonstrated absorption maxima within the tested frequency range) and hybrid CF–ferrite- or CB–ferrite-based composites, one can see that composites filled only with ferrites exhibited the highest matching frequencies f*_m_* and the broadest effective absorption frequency bandwidths Δf at −10 and −20 dB. Based upon that, it can be concluded that those composites are the best EMI absorber shields. Simultaneously, they can shield electromagnetic radiation at the highest frequencies. The incorporation of carbon-based fillers resulted in the shifting of absorption shielding performance of composites to lower frequencies on one hand. On the other hand, their absorption shielding performance was lower as evidence of lower effective frequency bandwidths. The poorest ability to absorb EMI was demonstrated by the composites filled with ferrites and carbon black, showing the narrowest absorption peaks. They also provided absorption shielding performance at the lowest frequencies. It can be stated that absorption maxima RL*_min_* were not significantly influenced by the composition of composite materials. 

**Figure 15:**
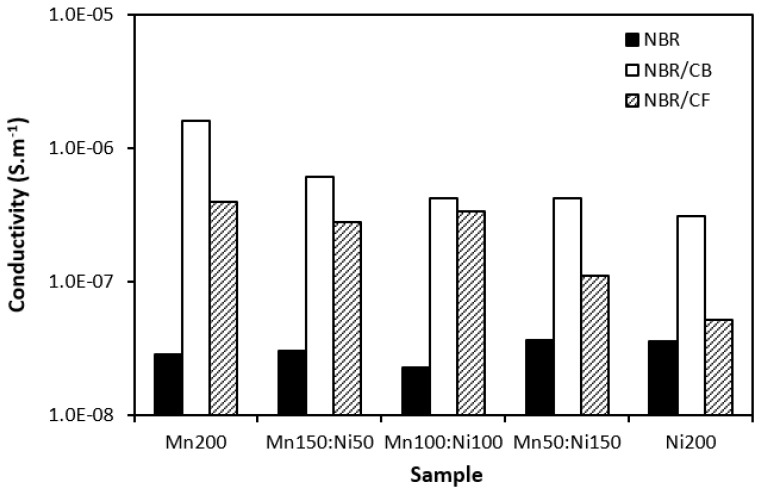
Influence of fillers on conductivity of composites.

The electrical conductivity of composite shields was evaluated to understand the influence of the fillers on absorption shielding performance. From Figure 15, it is obvious that the lowest conductivity was found in composites filled only with ferrites. Carbon-based fillers demonstrate unique electrical properties and it becomes clearly apparent that their application into rubber compounds results in an increase in electrical conductivity. The highest electrical conductivity was found for composites filled ferrites and carbon black. The conductivity of hybrid composites was found to increase with an increasing content of manganese–zinc ferrite, which could point to s higher conductivity of MnZn filler. However, this was not experimentally confirmed for composites filled only with ferrites. As shown, their conductivity moved only in a very low range of experimental values with almost no dependence on the type of ferrite or ferrites in their combinations. It might be stated that some synergic effect between carbon-based fillers and ferrites was observed when considering the conductivity. The highest conductivity was manifested in the composite CB-Mn200. This composite simultaneously showed the highest real and imaginary permittivity values (Figure 11). With increasing proportions of NiZn ferrite, the conductivity of both types of hybrid composites showed decreasing trends and similar decreases were observed for their permittivity. Also, the highest conductivity of composites filled with ferrites and CB was reflected in their highest real and imaginary permittivity values. On the other side, the lowest permittivity was demonstrated for the composites filled only with ferrites with the lowest conductivity. As their conductivity was not significantly influenced by the type of ferrite, we recorded the lowest difference between the real and imaginary permittivity values for composites Mn200 and Ni200. Thus, the dependence between the conductivity and permittivity was established, meaning that higher conductivity was reflected in the higher complex permittivity of composites. As outlined, real permittivity relates to the electrical charge storage capacity. It can be measured as the number of accumulated charges, microcapacitors, and polarization centers [18,26]. Polarization of the filler and rubber matrix and polarization at the interfacial region between the filler and the matrix can occur in dependence of radiation frequency [27,28]. The presence of carbon-based fillers resulted in higher accumulation of electric charges within the composite materials. Simultaneously, the application of carbon-based fillers caused a reduction in the distance between the filler particles, which are surrounded by the rubber matrix. Even a small content of carbon-based fillers significantly reduces space between particles due to the tubular structure of carbon fibers or structural aggregates of carbon black. This led to higher polarization of the rubber matrix as well as formation of localized charges at the filler–rubber interfacial region (interfacial rubber–filler polarization). Imaginary permittivity relates to the dissipation of electrical energy. The presence of carbon-based fillers contributed to the formation of conductive networks within the composite matrix, which is beneficial for dielectric dissipation and, thus, higher imaginary permittivity [29,30].

Although, at lower frequencies (below 1 GHz), slightly higher real and imaginary permeability values were exhibited in hybrid composites, overall, it be stated that the permeability of composites was found not to be significantly influenced by the type and content of the fillers. Real and imaginary permeability corresponds to magnetic storage and dissipation, respectively. As shown, the complex permeability is influenced only by the presence of magnetic fillers with magnetic dipoles with almost no influence of highly conductive carbon-based fillers. Materials having magnetic dipoles and, thus, high permeability have been reported to be good EMI absorber shields [31,32,33]. However, all tested composites exhibited very similar permeability and higher permittivity, and conductivity of composites containing carbon-based fillers are believed to be crucial parameters, which diminished their absorption shielding performance. It has been revealed that highly conductive materials are good candidates for reflection of electromagnetic radiation, mainly at low frequencies [34,35,36]. The results also demonstrated that the higher the conductivity, the lower the frequency for absorption shielding. 

### 3.3. Physical–Mechanical Characteristics

**Figure 16:**
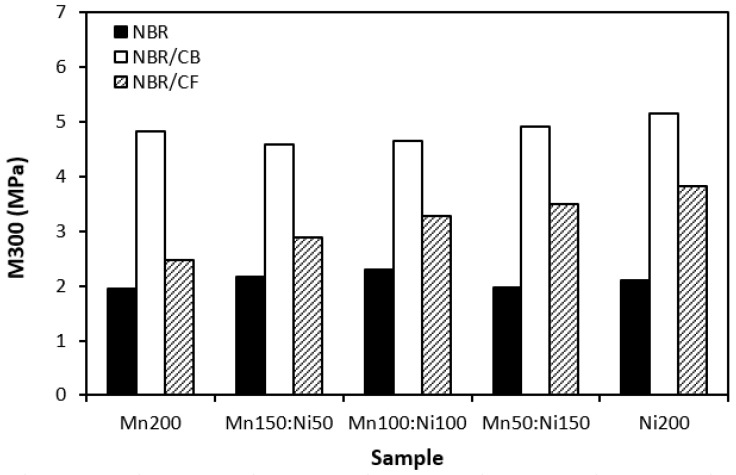
Influence of fillers on modulus M300 of composites.

**Figure 17:**
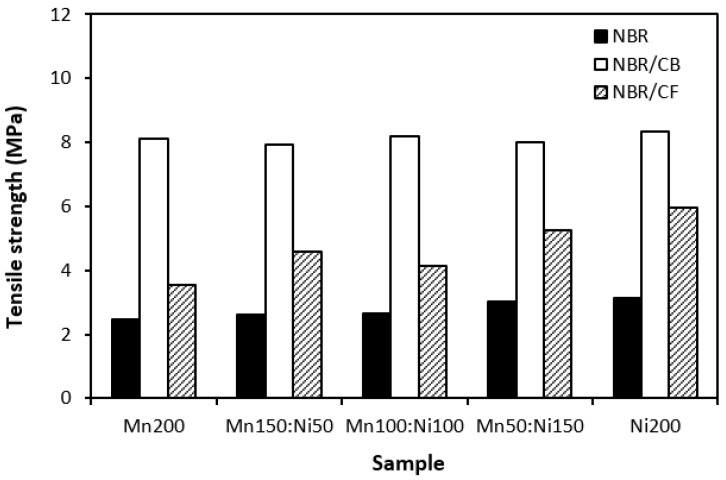
Influence of fillers on tensile strength of composites.

**Figure 18:**
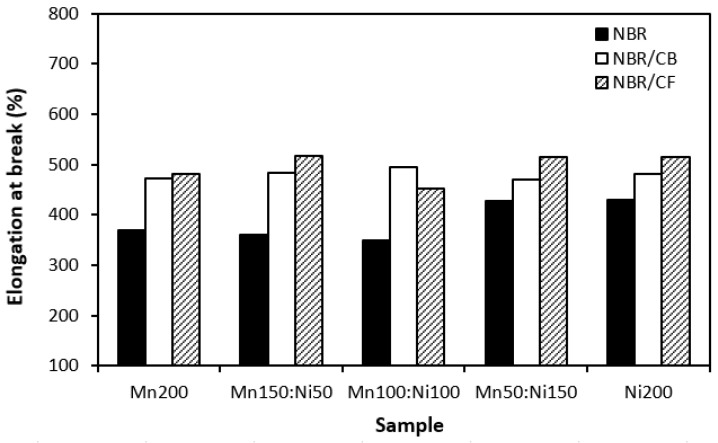
Influence of fillers on elongation at break of composites.

**Figure 19:**
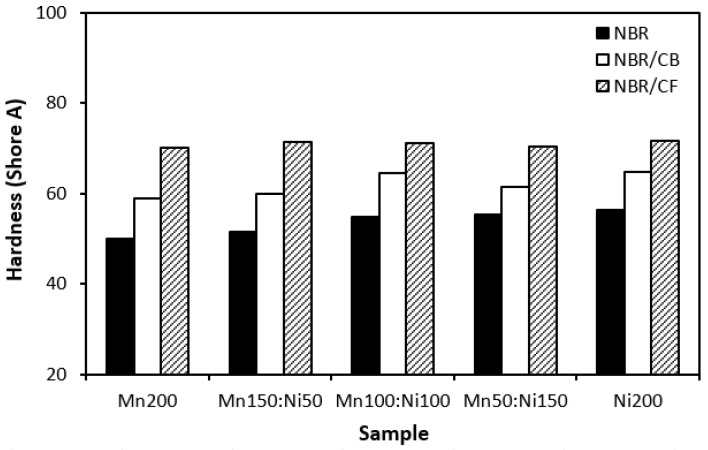
Influence of fillers on hardness of composites.

Physical–mechanical properties were investigated to determine the utilization of tested materials in practical applications. Looking at Figure 16 and Figure 17, one can see very similar dependences of modulus M300 and tensile strength on the tested fillers. The lowest modulus (Figure 16) and tensile strength (Figure 17) were demonstrated for the composites filled with magnetic fillers. This is a logical reflection of the fact that ferrites as stiff powdery fillers do not act as reinforcing fillers when they are incorporated into a rubber matrix. By incorporation of carbon-based fillers, both modulus and tensile strength increased. The highest values of both characteristics were manifested in the composites filled with a combination of ferrites and carbon black. CB is the most widely used nano-filler in rubber technology with its aggregated structure providing enhanced tensile behavior to rubber materials. As shown in Figure 17, the tensile strength increased from about 2.5–3 MPa for composites filled only with ferrites to roughly 8 MPa for composites with incorporated carbon black. The application of carbon-based fillers caused increases in elongation at break and the hardness of composites (Figure 18 and Figure 19)., The highest values of both properties were exhibited in composites filled with ferrites and carbon fibers. However, it seems that the modulus and tensile strength of hybrid ferrites and CF-filled composites showed increasing tendency with an increasing ratio of NiZn filler; in general, it can be stated that no significant changes in physical–mechanical characteristics were observed in dependence on the type of ferrite or ferrites in their combinations. 

## 4. Conclusions

Rubber composites based on magnetic fillers and the combination of magnetic fillers with carbon-based fillers were examined for their curing process, physical–mechanical properties and EMI absorption shielding performance. The results showed that curing characteristics were not dependent on the type of ferrite or ferrite combination. The application of carbon black and carbon fibers resulted in the acceleration of the curing kinetics, as carbon-based fillers enhanced thermal flow through the materials and enabled them to be heated faster up to the curing temperature. Similarly, no influence of the type of ferrite or magnetic fillers in their combinations on physical–mechanical characteristics was recorded. The incorporation of carbon-based fillers caused the enhancement of physical–mechanical properties. The highest modulus and tensile strength were exhibited in composites filled with ferrites and carbon black, while the highest hardness and elongation at break manifested in composites with ferrites and carbon fibers. The presence of carbon-based fillers in rubber compounds led to an increase in their conductivity and permittivity. The conductivity and permittivity of composites increased in the following order: ferrites < CF/ferrites < CB/ferrites. The higher the conductivity and permittivity, the lower the absorption shielding performance. The absorption shielding efficiency of composites increased as follows: CB/ferrites < CF/ferrites < ferrites. The frequency for absorption shielding increased in the same order. The higher the proportion of nickel–zinc ferrite in magnetic filler combinations, the lower the conductivity and permittivity, suggesting that nickel–zinc ferrite provided better absorption shielding performance. 

## Figures and Tables

**Table 1:** The particle size distributions of magnetic fillers.

Filler	Particle Size Distribution	D10	D50
MnZn ferrite	0.7–50 µm	4.7 µm	16.3 µm
NiZn ferrite	0.2–70 µm	3.0 µm	21.4 µm

**Table 2:** The composition of materials filled with ferrites in phr.

NBR	100	100	100	100	100
ZnO	3	3	3	3	3
stearic acid	2	2	2	2	2
CBS	1.5	1.5	1.5	1.5	1.5
sulfur	1.5	1.5	1.5	1.5	1.5
MnZn ferrite	200	150	100	50	0
NiZn ferrite	0	50	100	150	200
designation	Mn200	Mn150	Mn100	Mn50	Ni200
		Ni50	Ni100	Ni150	

**Table 3:** Composition of materials filled with carbon-based fillers and ferrites in phr.

NBR	100	100	100	100	100
ZnO	3	3	3	3	3
stearic acid	2	2	2	2	2
CBS	1.5	1.5	1.5	1.5	1.5
sulfur	1.5	1.5	1.5	1.5	1.5
CB or CF	25	25	25	25	25
MnZn ferrite	200	150	100	50	0
NiZn ferrite	0	50	100	150	200
designation	CB, CF-Mn200	CB, CF-Mn150	CB, CF-Mn100	CB, CF-Mn50	CB, CF-Ni200
		Ni50	Ni100	Ni150	

**Table 4:** Absorption characteristics of materials filled with ferrites.

Sample	RL*_min_*(dB)	f*_m_*(MHz)	Δf (MHz) −10 dB	Δf (MHz) −20 dB
Mn200	−60.1	4670	2950	1100
Mn150Ni50	−51.4	5700	2450	980
Mn100Ni100	−31.1	6000	2300	630
Mn50Ni150	20.3	6000	2800	200
Ni200	−9.7	6000	-	-

**Table 5:** Absorption parameters of materials filled with ferrites and carbon fibers.

Sample	RL*_min_*(dB)	f*_m_*(MHz)	Δf (MHz) −10 dB	Δf (MHz) −20 dB
CF-Mn200	−58.3	2769	2130	600
CF-Mn150Ni50	−72.6	3250	2500	720
CF-Mn100Ni100	−49.2	3820	3140	900
CF-Mn50Ni150	−54.8	4860	2980	1130
CF-Ni200	−57.0	4140	3600	1380

**Table 6:** Absorption parameters of materials filled with ferrites and carbon black.

Sample	RL*_min_*(dB)	f*_m_*(MHz)	Δf (MHz) −10 dB	Δf (MHz) −20 dB
CB-Mn200	−49.0	1780	1060	300
CB-Mn150Ni50	−49.5	2180	1500	430
CB-Mn100Ni100	−47.8	2660	2240	600
CB-Mn50Ni150	−59.0	2880	2500	700
CB-Ni200	−51.0	3250	3100	880

## Data Availability

Data are contained within the article.
